# State-Space Model for Arrival Time Simulations and Methodology for Offline Blade Tip-Timing Software Characterization

**DOI:** 10.3390/s23052600

**Published:** 2023-02-26

**Authors:** Tommaso Tocci, Lorenzo Capponi, Gianluca Rossi, Roberto Marsili, Marco Marrazzo

**Affiliations:** 1 Department of Engineering, University of Perugia, Via G. Duranti 93, 06125 Perugia, Italy; 2 Baker Hughes, Via F. Matteucci 2, 50127 Florence, Italy

**Keywords:** tip-timing, diagnostics, blade vibration, turbomachinery, state-space, arrival time, aeromechanics

## Abstract

Blade tip-timing is an extensively used technique for measuring blade vibrations in turbine and compressor stages; it is one of the preferred techniques used for characterizing their dynamic behaviors using non-contact probes. Typically, arrival time signals are acquired and processed by a dedicated measurement system. Performing a sensitivity analysis on the data processing parameters is essential for the proper design of tip-timing test campaigns. This study proposes a mathematical model for generating synthetic tip-timing signals, descriptive of specific test conditions. The generated signals were used as the controlled input for a thorough characterization of post-processing software for tip-timing analysis. This work represents the first step in quantifying the uncertainty introduced by tip-timing analysis software into user measurements. The proposed methodology can also offer essential information for further sensitivity studies on parameters that influence the accuracy of data analysis during testing.

## 1. Introduction

When forced vibrations occur at (or near) the natural frequencies of compressor and turbine stage blades, fatigue effects can cause mechanical failure [1]. Therefore, effective diagnostic tools to monitor blade dynamics are crucial for proper turbomachinery component design [2,3]. While strain gauges historically validated rotating blade dynamics, they have limited lifetimes in high-temperature environments and provide limited information on instrumented blades [4,5,6]. To overcome the intrusiveness of strain gauges and their complexity in transmitting data from a rotating system, the blade tip-timing (BTT) technique has become one of the most advanced and versatile techniques for axial turbomachinery blade dynamics measurements [2,3]. BTT is a non-contact technique that uses probes installed on the engine casing [7,8] to measure the time of arrival of each blade. In the absence of blade vibration, the time of arrival would ideally be equal to the expected values for a rotating rigid blade, determined by the rotor and blade geometry and the system’s dynamics. However, in the presence of vibrations, an actual series of times-of-arrival is measured [9,10], and the difference between the measured and expected arrival time is used to calculate the displacement of each blade tip in the rotation plane [11,12].

In general, blade tip-timing is an experimental technique that allows for the determination of the deflection of the blades of turbines or compressors from the measured delays.

Recently, many works related to blade tip-timing have been presented. Mohamed et al. [13] presented the process for validating the FE stress and deflection predictions of aero-engine compressor blades under non-rotation conditions as a critical preliminary step toward a complete understanding of their dynamic behavior under rotating conditions when using the BTT measurement. Przysowa et al. [14] utilized BTT for the forced vibration analysis of axial compressor blades. Wei et al. [15] developed a blade tip-timing signal simulator based on a novel model reduction method. Bornassi et al. [16] performed BTT measurements of transient vibrations in mistuned-bladed disks operating in non-stationary conditions. Tchuisseu et al. [17] developed a method for optimizing probe positioning in blade tip-timing systems.

The BTT measurement technique involves the use of different types of probes that vary according to the specific case, e.g., laser probes [1,18,19], microwave sensors [20], magnetoresistive sensors [21,22], capacitive sensors, [23] and magnetic pickup sensors. However, optical probes are usually preferred for their accuracy and resolution [24,25,26,27,28]. Besides the specific nature of the sensing probe, the arrival time signals are usually acquired and processed with a dedicated BTT measurement system. At the industrial level, it is common to use commercial software for handling the elaboration of tip-timing data. Typically, such software adopts a “black-box” model, where it is not possible for the user to completely understand how the data are handled and processed. A complex set of acquisition and data processing parameters need to be set to obtain reliable information on the system’s dynamics.

Therefore, it is essential for the operator to gain experience in using such software. Consequently, the output of such an analysis is often influenced by how the various parameters, made available by the BTT analysis software, are managed. Therefore, this work represents the first step in quantifying the uncertainty that tip-timing analysis software introduces into the measurement by users.

The present work is part of a larger project aimed at characterizing the entire tip-timing measurement chain from a software and hardware perspective. Capponi et al. [29] published the first work in this direction and conducted an experimental investigation on the hardware and triggering effects in tip-timing measurement uncertainty.

The proposed methodology can also provide fundamental information for further sensitivity studies on the parameters that influence the accuracy of the analysis of the data acquired during testing. In addition, this type of approach makes it possible to outline an objective methodology for choosing the analysis parameters, knowing the influence on the measurement uncertainty. This research is based on the sensitivity analysis of data processing parameters and signal conditioning techniques typically used for the time-of-arrival analysis in common BTT software. For this purpose, this study proposes an innovative mathematical model for generating synthetic arrival time signals. While the models implemented throughout the years for the simulation of BTT measurements mostly involve the finite element method analysis [30,31], the authors propose a state space-based approach for generating simulated arrival time signals, controlling geometry, and dynamics of turbomachinery rotating blades (e.g., rotational frequencies, resonance amplitudes, and signal noise components). These arrival time signals are used as the controlled input for the sensitivity analysis of a common BTT software for the blade tip-timing type analysis. The known amplitude and resonant frequency value were imposed on the blade deflection. The performance of the software was evaluated as its parameters changed by comparing the output with the characteristics imposed upstream on the deflection by our mathematical model.

At this stage, only synchronous vibrations of the blades with a single vibration mode were simulated. The manuscript is organized as follows: Section 2 describes the mathematical model and explains which parameters of the BTT software are tested through the simulated synthetic data. Section 3 reports the results obtained during the analysis of the signals generated using the software. In Section 4, the results are discussed, and an example of software information that can be inferred from the simulated data sets is given. Section 5 draws the conclusions.

## 2. Material and Methods

### 2.1. BTT Fundamentals

A BTT measurement system setup includes a number of non-contact sensors placed on the casing of a turbomachine. The placement and spacing of the sensors are determined by the relevant natural frequencies over a specified speed range, as well as the expected engine orders and mode shapes [29,32]. The BTT technique relies on measuring the time of arrival, i.e., the moment when the blade passes in front of each sensor. The time of arrival for a rigid blade, $t_{r}$ (i.e., a blade without vibration), is specified as [29,32]:

(1) $$t_{r}=\frac{\theta }{\omega }$$

where θ represents the angular distance covered by the blade for passing from the reference’s angular position to the probe location and ω refers to the angular velocity of the shaft. A blade with vibration will interact with the sensor beam at different time intervals compared to a purely rigid blade with a constant time gap, Δt, due to the effect of vibration on the steady blade motion. The time of arrival for a vibrating blade, $t_{v}$, is as follows [29,32]:

(2) $$t_{v}=t_{r}\pm \Delta t$$

The time gap Δt is used by the BTT system to determine the deflection of the blade tip, δ [29,32]:

(3) δ=ωRΔt

where *R* is the radius at the point measured along the blade. Since the sampling rate is given by the blade passing in front of the probes, in real applications, the vibration sinusoid is significantly under-sampled. This requires dedicated software for post-processing arrival times and derives blade vibration characteristics.

### 2.2. Algorithm for Transient Arrival Time Simulation

The aim of this algorithm is to generate a series of arrival times, using efficient mathematical formulations, simulating a turbine ramp acceleration between two fixed speed values. However, this approach requires using a velocity range such that the vibration cycle is completely developed and it has at the peripheries of the ramp zero deflection. For this purpose, nominal arrival times were generated without imposing any vibration on the rotor blades. Then, blade vibrations with known amplitudes and frequencies were superimposed on the generated arrival time. This was done by simulating vibration with the resonance of the mechanical system using a state-space modeling technique [33]. The resonance was placed in the middle of the simulation interval. In addition, a check was made to the output file, calculating the amplitude and frequency of the deflection starting from the calculated arrival time and comparing it with the imposed values. The angular velocity ω(t) is defined as:

(4) $$\omega \left(t\right)=\omega_{s}+a_{r}\cdot t=\omega_{s}+\left(\frac{\omega_{f}-\omega_{s}}{T}\right)\cdot t,$$

where *t* is a time value within the duration of the simulation *T*, $\omega_{s}$ is the initial angular velocity [rad/s], $\omega_{f}$ is the final angular velocity [rad/s], and $a_{r}$ is the angular acceleration $\left[rad/s^{2}\right]$. The location of the angular blade $\theta_{b}\left(t\right)$ is defined as:

(5) $$\theta_{b}\left(t\right)=\theta_{b0}+\int \omega \left(t\right)\;\mathrm{dt},$$

where $\theta_{b0}$ is the starting angular position of the blade. Substituting Equation (4) in Equation (5), as well as integrating, it follows that

(6) $$\theta_{b}\left(t\right)=\theta_{b0}+\omega_{s}\cdot t+\frac{1}{2}\cdot \left(\frac{\omega_{f}-\omega_{s}}{T}\right)\cdot t^{2}.$$

The array of nominal arrival times is determined by imposing that the angular position of the blade $\theta_{b}$ coincides with the angular position of the probe $\theta_{p}$, as shown in Equation (7), where *k* is an integer parameter that indicates the round number. A time instant *t* is defined as the *arrival time* when it is a solution of:

(7) $$\theta_{b0}+\omega_{s}\cdot t+\frac{1}{2}\cdot \left(\frac{\omega_{f}-\omega_{s}}{T}\right)\cdot t^{2}-2k\pi =\theta_{p}.$$

**Figure 1:**
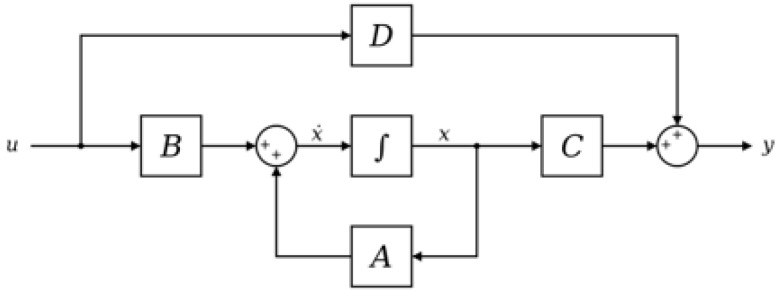
Graphical representation of the state-space system.

The vector of nominal arrival times is obtained by solving Equation (7) for all blades, all probes, and each revolution. To superimpose the blade deflection on the previously calculated nominal arrival times, the vibrating system of the blade, assumed to be a damped forced vibration [34], needs to be modeled. However, the angular acceleration component that takes the rotational velocity from the initial values to the final values must be taken into account. This causes the rotational regime to be transient; therefore, the commonly used equations for damped forced vibrations used under steady-state conditions cannot be applied [34]. For this purpose, a *state-space* approach is selected. The state-space representation of a system replaces a differential equation of order *n*th with a single first-order matrix differential equation, greatly simplifying the solution of the mechanical system [34]. The state-space representation of a system is shown in Figure 1 and is given by:

(8) $$\dot{q}\left(t\right)=A\;q\left(t\right)+B\;u\left(t\right),$$

(9) y(t)=Cq(t)+Du(t).

Equation (8) is called the *state* equation, while Equation (8) is the *output* equation. For a system of the *n*th order, with *r*-inputs and *m*-outputs, and dimensions of arrays in brackets, it follows that *q* is the state vector and is a function of time (n × 1 dimension − n rows by 1 column). Moving on the matrices, *A* is the state matrix (n × n), *B* is the input matrix (n × r), *C* is the output matrix (m × n), and *D* is the direct transition (or feed-through) matrix (m × r). Finally, the inputs and outputs are defined, both of which are functions of time; *u* is the input (r × 1) and *y* is the output (m × 1).

**Figure 2:**
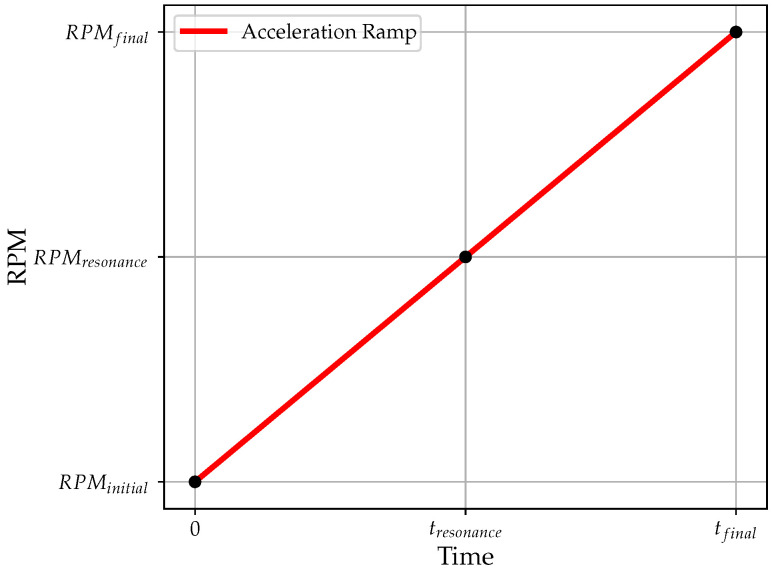
Acceleration ramp: the $RPM_{initial}$ is the starting angular velocity and $RPM_{final}$ is the angular velocity reached at the end of the ramp.

The resonance frequency is calculated, beginning with the rotation regime, using the mean angular velocity of the simulation and multiplying it by the chosen per-rev model, as shown in Figure 2 and Equation (11). Typically, a monotonic ramp is considered for the tip-timing analysis. In this work, a ramp with constant acceleration was considered for simplicity, as shown in Figure 2. However, different types of ramps can be applied by simply replacing the acceleration equation. Note that the per-rev model corresponds to the Campbell diagram order number.

The resonance angular velocity is determined as follows:

(10) $$RPM_{resonance}=\frac{RPM_{final}-RPM_{initial}}{2}.$$

The resonance frequency depends on the chosen per-rev model and is calculated through:

(11) $$f_{r}=\frac{RPM_{resonance}}{60}\cdot xr.$$

The xr parameters are chosen to excite a specific engine order and, consequently, ensure that the chosen per-rev model is that of the best data fitting.

**Figure 3:**
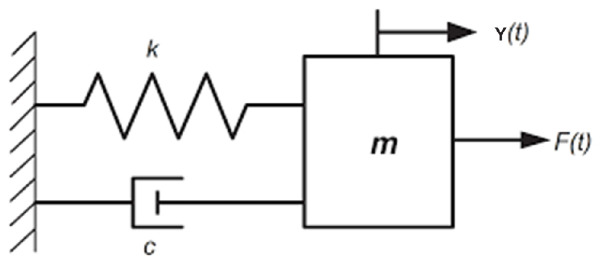
Damped forced vibration system.

Each blade is schematized as shown in Figure 3, where *m* is the blade mass, *k* is the blade stiffness, *c* is the damping constant, F(t) is the external force, and y(t) is the system output. Here, the external force is defined as a sine sweep, according to the following equation:

(12) F(t)=Asin(ω(t)·t)

Parameters $\omega_{r}$, *m*, *k*, and *c* are tuned to obtain the resonance at the desired frequency and the per-rev model:

(13) $$\left\{\begin{array}{c} \omega_{r}=2\pi f_{r}\cdot xr\\ \frac{k}{m}={\omega_{r}}^{2}\\ \frac{c}{m}=2\zeta \omega_{r} \end{array}\right.$$

where ζ is called...in relation to the mechanical system described in Figure 3 and Equations (8) and (9), the state-space matrices are defined as:

(14) $$A=\left[\begin{array}{cc} 0 & 1\\ \frac{-k}{m} & \frac{-c}{m} \end{array}\right];\;B=\left[\begin{array}{c} 0\\ \frac{1}{m} \end{array}\right];\;C=\left[1,0\right];\;D=\left[0\right],$$

The system response is determined using the linear system simulation toolbox. This application requires (as input parameters) the forcing function and the state-space matrices. From the simulated full response signal, the responses at the instants corresponding to the nominal arrival times are extracted. Using the extracted response values, a new response vector disp(t) is built. Regarding the procedure for applying deflection to arrival times, a Δt time variation is added to each element of the previously calculated arrival time array (at) by:

(15) $$t_{D}=t+\Delta t\left(t\right),$$

where $t_{D}$ is the arrival time with deflection, *t* is the nominal arrival time, and Δt(t) is the variation in time due to the deflection effect. The function Δt(t) is defined as follows:

(16) $$\Delta t\left(t\right)=\frac{disp(t)}{\omega (t)R}$$

The arrival time arrays generated are encoded in binary files compatible with the BTT software used in this research.

### 2.3. Arrival Time Array Validation through Deflection Calculation

A preliminary validation of the developed algorithm is performed. Taking into account a single blade (identified with an identification number id) and all arrival times produced by it on all probes, two arrays are produced: one containing all nominal arrival times t(id), and one with deflection $t_{D}\left(id\right))$. For each i-*th* element of this matrix, the time variation Δt(id) is recalculated:

(17) $$\Delta t\left(id,i\right)=t_{D}\left(id,i\right)-t\left(id,i\right).$$

Finally, the deflection is determined with Equation (18) and plotted in the time and frequency domains, using the fast Fourier transform (FFT) method:

(18) $$S\left(t\right)=\Delta t_{c}\left(id\right)\cdot \omega \left(t\right)\cdot R.$$

Under the condition of completely equispaced probes, the BTT sample frequency is equal to the product of the angular velocity RPM and the number of probes $n_{p}$:

(19) $$f_{s}=\frac{RPM}{60}\cdot n_{p}$$

In order to validate the simulated signal generation algorithm, the resonant frequency and amplitude were imposed. By analyzing the signal using frequency-based methods, such as the fast Fourier transform (FFT), it was expected to measure the same frequency and amplitudes imposed initially. If the analysis is not affected by aliasing, applying a simple FFT would allow us to verify the quality of the generation. Assuming an angular velocity of 9250 RPM and a number of probes $n_{p}$ = 8, a sampling frequency $f_{s}\approx$ 1233 Hz is obtained through Equation (19).

**Figure 4:**
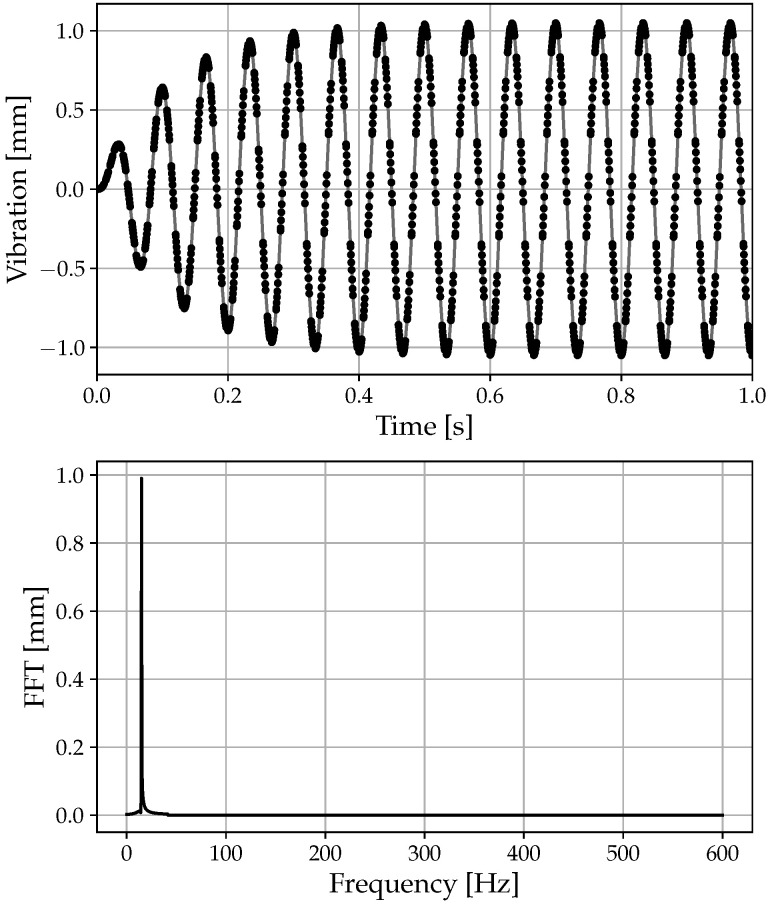
Example of deflection: 9000 to 9500 RPM, $f_{r}$ = 15 Hz, A = 1 mm: amplitude response and deflection FFT.

In Figure 4, it is possible to see how the response perfectly matches the imposed input in both the frequency content and amplitude. In this case, given the resonance frequency $f_{r}$ = 15 Hz, the resonance amplitude $A_{R}$ = 1 mm, and the sampling frequency $f_{s}\approx$ 1233 Hz, the complete absence of the aliasing phenomenon is highlighted. By analyzing the FFT signal, it is evident that the identified frequency and its amplitude are completely coincident with those imposed. In fact, examining the FFT signal reveals a 15 Hz frequency peak with an amplitude of 1 mm, which matches the specified amplitude exactly.

**Figure 5:**
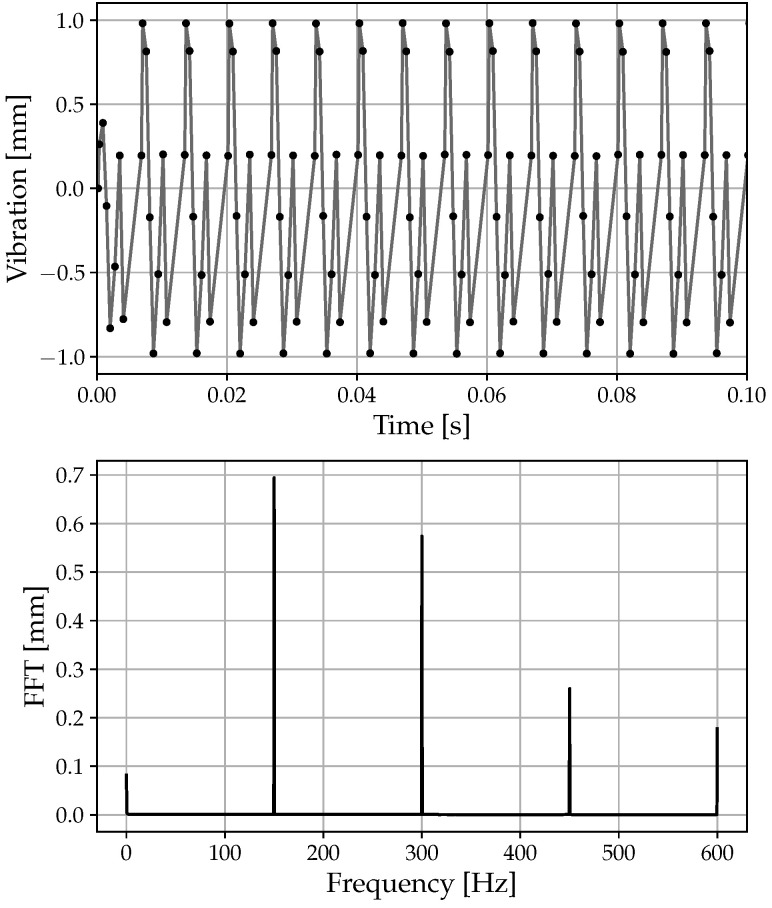
Example of deflection: 9000 to 9500 RPM, $f_{r}$ = 1500 Hz, A = 1 mm: amplitude response and deflection FFT.

In Figure 5, an example of a high resonance frequency computation that produces the aliasing phenomenon is given. In this case, the aliasing phenomenon is evident as the sampling frequency ($f_{s}\approx$ 1233 Hz) is smaller than the resonance frequency ($F_{r}$ = 1500 Hz). In contrast to the previous case, the imposed resonance frequency cannot be identified in the FFT due to strong aliasing. Despite this, sub-harmonics of the imposed resonance frequency (1500 Hz) are visible. Since the BTT software deals with strong aliasing, this is typical in real applications.

The validation procedure for the generation algorithm is now reported. It is based on data analysis with dedicated BTT software. The data were generated with resonance parameters similar to those relevant to real-world applications, and only the case of aliasing is reported as the most unfavorable scenario.

Sets of signals with parameters similar to those previously seen were generated, where the sampling frequency was approximately 1233 Hz. Synthetic signals were generated by alternately fixing either the resonant frequency $F_{r}$ or the resonant amplitude $A_{r}$, and the percentage difference between the imposed value and the measured value in terms of frequency ($\Delta F_{r}$) and amplitude ($\Delta A_{r}$) was verified. In Table 1, the results for a resonance amplitude fixed at 0.05 mm and a resonance frequency ranging from 1500 to 30,000 Hz are shown. Analyzing the results, a maximum difference in terms of frequency is obtained to be less than 0.03% and in terms of the amplitude of 4%, but under high resonance frequency conditions. The results obtained are considered entirely acceptable, showing a perfect match between the set and measured parameters.

Finally, in Table 2, the analysis of signals generated with a constant resonance frequency (10,000 Hz) and amplitude ranging from 0.05 to 0.5 mm is shown. In this case, results similar to those seen in the previous case are also evident. The combination of results in Table 1 and those in Table 2 shows the low uncertainty in the model developed and then its validity for the intended use.

### 2.4. Random Noise

In order to simulate pseudo-real test cases, random noise is added to the blade deflection signal. Referencing Equation (16) for the calculation of the time variation due to the superimposed deflection on the nominal arrival time, a component due to noise is added. Therefore, the new Δt equation is:

(20) $$\Delta t\left(t\right)=\frac{disp\left(t\right)+rnd\cdot A_{n}}{\omega (t)R},$$

where rnd is a random float number from 0 to 1, and $A_{n}$ is the noise amplitude. $A_{n}$ is calculated as shown in Equation (21), where xn is the random noise multiplier:

(21) $$A_{n}=A_{r}\cdot xn$$

**Figure 6:**
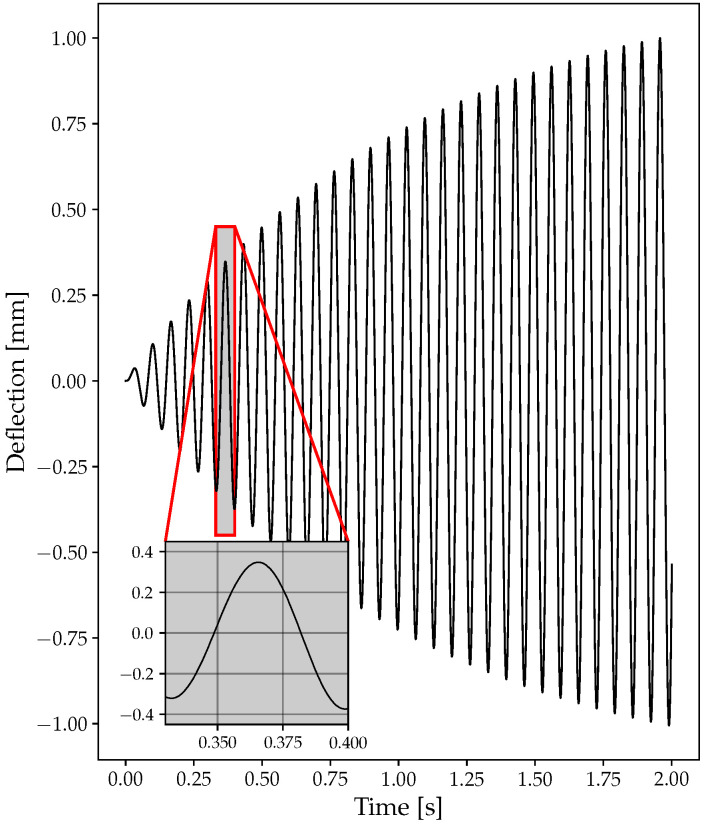
Example of deflection without noise: 9000 to 9500 RPM, *fr* = 154.2 Hz, A = 1 mm.

**Figure 7:**
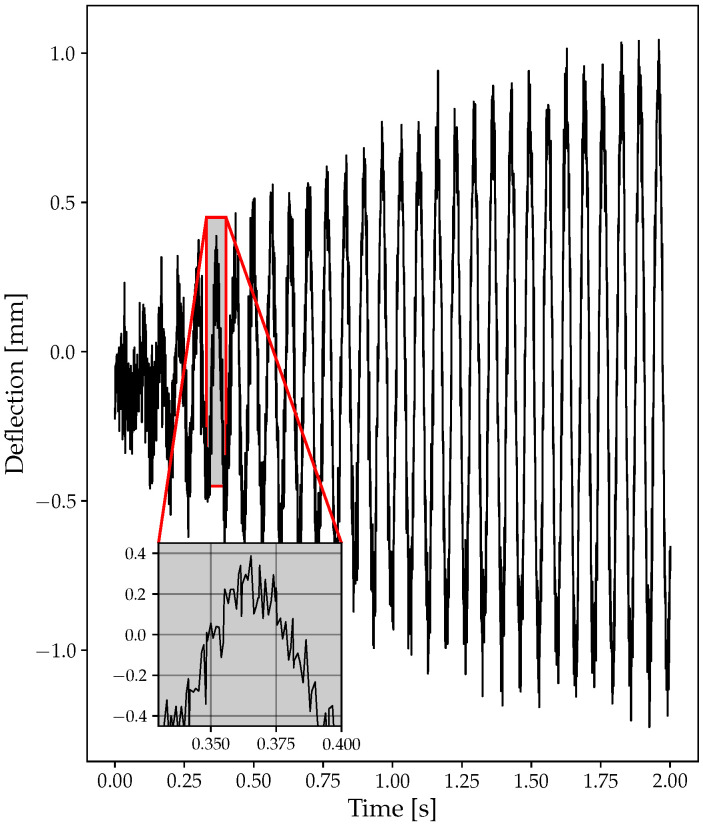
Example of deflection with noise: 9000 to 9500 RPM, *fr* = 154.2 Hz, A = 1 mm. The random noise amplitude is equal the 10% of resonance amplitude A.

For illustration purposes, two sample deflection signals are shown. Both signals refer to a simulation with a ramp from 9000 to 9500 RPM with an angular acceleration of 3000 RPM/min. The signal was generated assuming the presence of 4 probes, 20 blades, and a sampling frequency of 100 KHz. The resonance frequency is 154.2 Hz with a resonance amplitude of 1 mm. Figure 6 shows an example of the signal generated without the addition of random noise. Figure 7 shows the signal with random noise added with an amplitude of 10% of the nominal resonance amplitude. The noise was added using Equation (21) and the relative deflection was calculated through Equation (18).

### 2.5. Analyzed Parameters

In this investigation, the parameters that were considered more relevant for the BTT analysis are as follows. The first parameter considered is *data fitting [%]*, which describes the quality of the interpolation between the vibration of the system and the model implemented in the BTT software. The second parameter considered is the *condition number (CN)*, which describes the quality of the location of the probe used during the experimental test. In our case, the location of the probe was a configurable parameter of the algorithm previously described (Section 2.2). The closer the condition number is to 1, the greater the ability of the software to reconstruct the blade vibration motion. The other parameters defined are ΔF and the ΔA. Δ*F* is the percent difference between the resonance simulated frequency and the frequency measured by the software as shown in Equation (22):

(22) $$\Delta F\left[\%\right]=\frac{\left|F_{software}-F_{resonance}\right|}{F_{resonance}}\cdot 100$$

Finally, *ΔA* is the percent difference between the resonance simulated amplitude and the amplitude measured by the software as shown in Equation (23):

(23) $$\Delta A\left[\%\right]=\frac{\left|A_{software}-A_{resonance}\right|}{A_{resonance}}\cdot 100.$$

**Figure 8:**
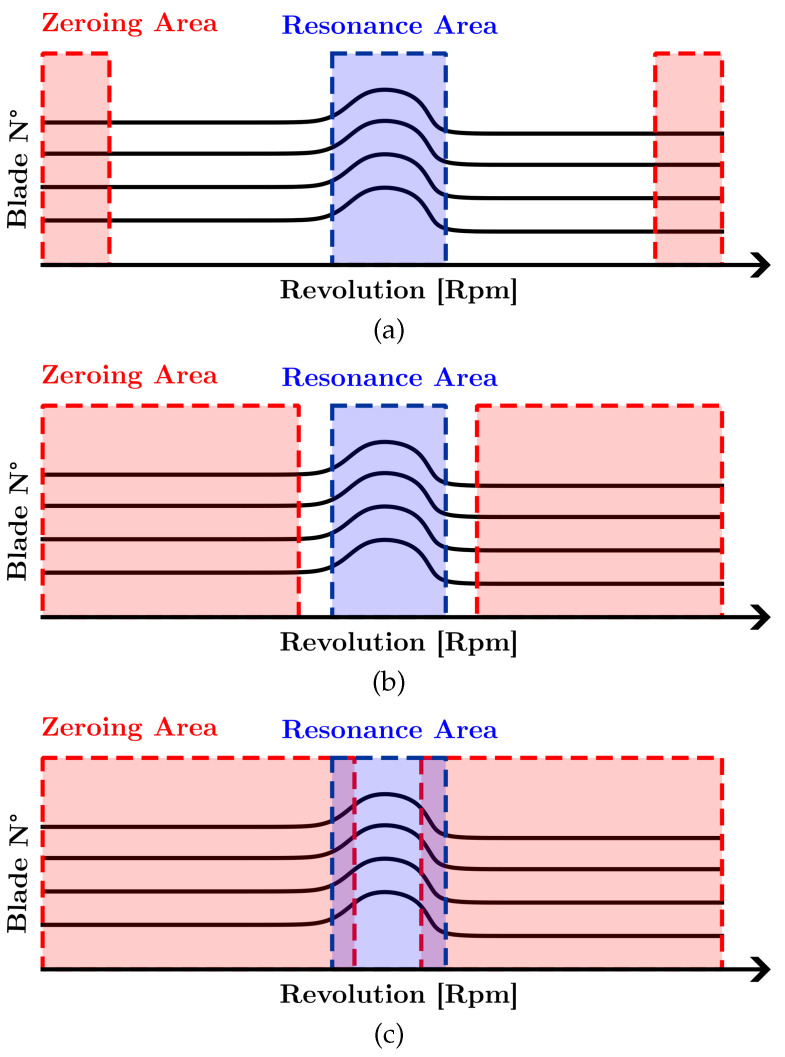
Scheme of the zeroing application. Deflections suffered by each blade (the plots are stacked vertically) as the number of revolutions (RPM) increases. The box in blue highlights the resonance area and red highlights the one chosen for the zeroing. Different types of zeroing are applied: (**a**) thin zeroing; the red zone is small and includes only the deflection at the lowest and highest RPM regime (**b**) large zeroing; the red zone is quite large, and includes deflection relative to wider RPM regions but it does not include part of the resonance area, (**c**) very large; the red zone is very large and it includes part of the resonance area.

It should be noted that the parameters considered here are generic, and each specific BTT software could present different options. In addition, it is necessary to point out that all of the analyses performed used the least mean square fitting (LSMF) method [35]. Moreover, regarding the simulation parameters that have a strong influence on the output, a few common settings usually have more influence on the output, i.e., the zeroing and low-pass filter (LPF). *Zeroing* is an essential procedure in the BTT signal analysis. In this study, the zeroing step is always performed with a zero-order polynomial but varies the signal portion used for the zeroing. Typically, three different types of zeroing are evaluated that depend on the size of the region under consideration. *Thin zeroing* is defined when a small extremal part of the signal (out of the resonance region) is used (Figure 8a). If a larger part is used instead, *large zeroing* or *very large zeroing* is defined. The difference is that, in the case of *large zeroing*, a large part of the signal is used but without including resonance areas (Figure 8b). Instead, with *very large zeroing*, a very large part of the signal is used, including resonance areas (Figure 8c). The *low pass filter (LP)* allows for the elimination of high-frequency noise from the signal. It needs to be used carefully because a higher value of the filter may also eliminate useful parts of the signal. The filters were tested using the following value of the filter LP: no filtering ($LP_{l}$), medium filtering ($LP_{m}$), and high filtering ($LP_{h}$). A high filtering level ($LP_{h}$) is defined as the maximum level that does not change the amplitude of the signal. The medium value ($LP_{m}$), on the other hand, is chosen as a middle ground between the unfiltered signal ($LP_{l}$) and the heavily filtered signal ($LP_{l}$).

### 2.6. Simulation Parameter

Different preliminary tests were carried out to determine the best linear simulation sampling frequency, considering the computation time and resolution. Other parameters, such as the per-rev, amplitude, and the noise multiplier, were tested in order to identify the most stressing set of parameters for the software. The simulation parameters selected in this study were: LSIM sample frequency = 1 MHz, resonance per-rev = 200, and the resonance amplitude = 0.05 mm. Four arrangements of eight probes returning different condition numbers were then evaluated. The angular combinations of the probes were evaluated with decreasing effectiveness as the condition number increased. In Table 3, the probe location values for each experimental set are shown.

In addition, different random noise components were added to the signal through the *noise multiplier* (NM) (Section 2.4). The values of NM applied are 10×, 50×, and 100×.

## 3. Results

The results obtained by comparing the test parameters imposed on the simulation and those measured using BTT software were analyzed. The influence of each analyzed parameter (Section 2.5) is shown individually. Then, the cross-influence between the different parameters is also evaluated.

### 3.1. Low-Pass Filter

The influences of the various levels of the low-pass filter on the fit of the data are firstly analyzed.

**Figure 9:**
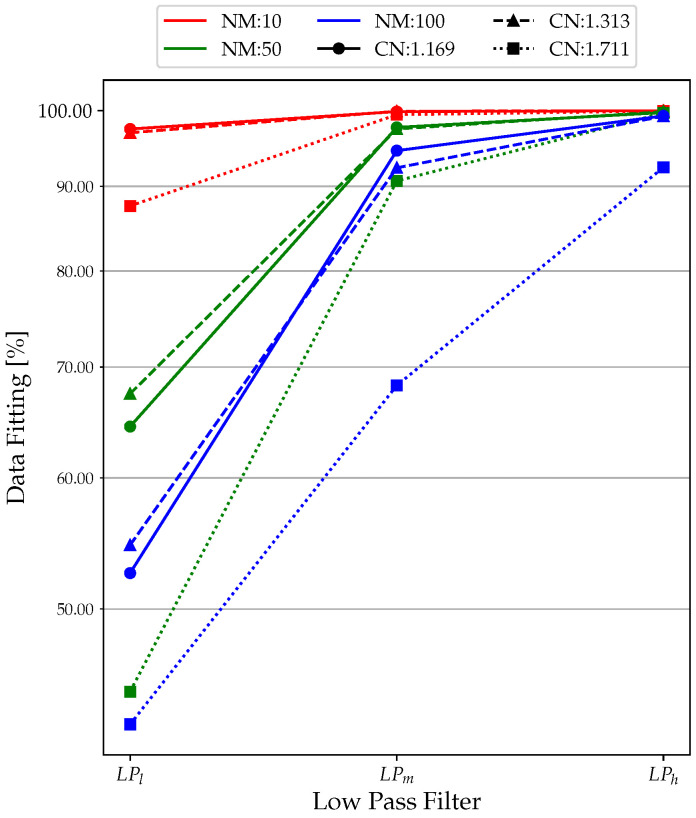
Low-pass filter analysis: data fitting results.

**Figure 10:**
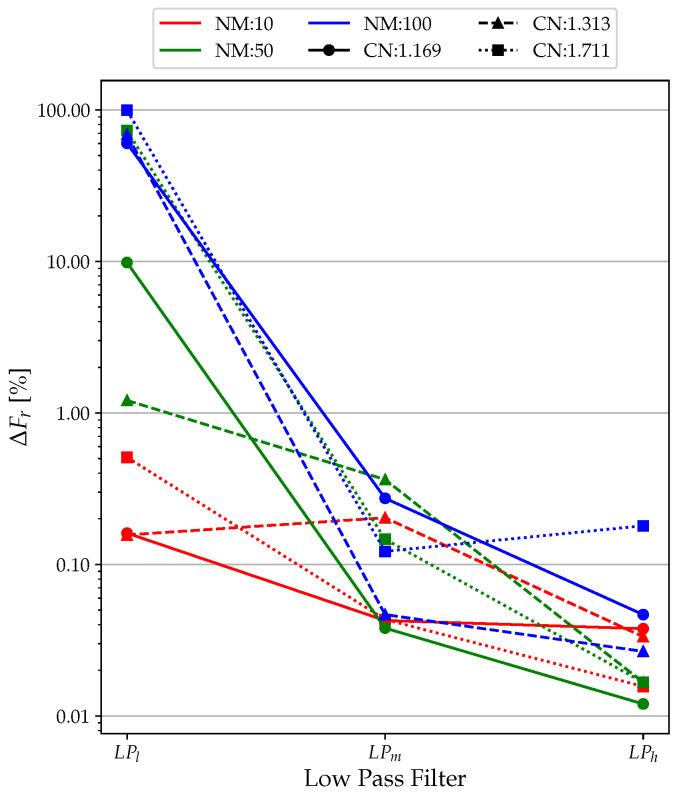
Low-pass filter analysis: Δ*F* results.

From Figure 9, the data fitting for LP${}_{l}$ performed poorly, with a minimum of just above 40% in the high CN and NM cases. The results improve for LP${}_{m}$, with almost all values above 90%. LP${}_{h}$ led to optimal results in terms of data fit, with all values close to 100% (minimum of 92% with NM = 100 and CN = 1.711). Clearly, the extremely unfavorable simulation conditions significantly reduce the performance of the low-pass filter. Similarly, in the case of Δ*F* (Figure 10), the no-filter condition (LP${}_{l}$ ) is efficient only for tests with NM = 10, far from the real experimental conditions. For NM = 50 and NM = 100, it can be observed that the values of Δ*F* oscillate between 10% and 100%, which are clearly unacceptable values.

**Figure 11:**
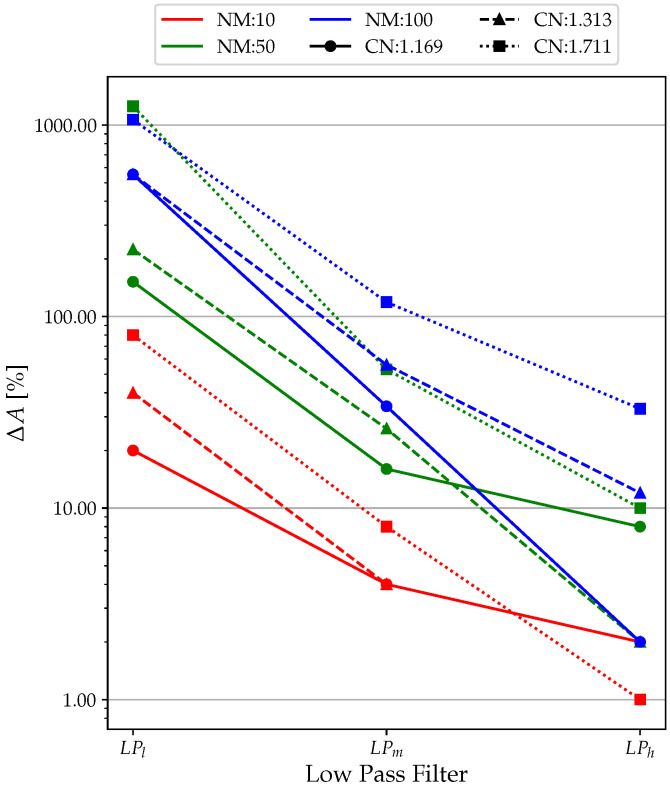
Low-pass filter analysis: Δ*A* results.

Using an LP${}_{h}$ filter reduces Δ*F* by about 5–10 times for each simulation, bringing it from a maximum value of 0.35% (NM = 50; CN = 1.313; LP${}_{m}$) to values completely below 0.050%. The only data that differ are that for NM = 100 and CN = 1.711, where the unfavorable simulation conditions make the filter inefficient, as can be seen from the data fitting. In terms of Δ*A*, it is possible to see the same trend obtained for Δ*F*, even with significantly higher errors, as shown in Figure 11. Here, the use of the LP filter is practically an obligatory choice as it allows one to contain the Δ*A* percentage to acceptable values. In fact, looking at the plots of Figure 8, the percent error in amplitude calculation reaches as high as 1000%, which is clearly unacceptable. This is due to the unfiltered signal that has amplitude values given by the noise, which increases the resonance, making it insidious to measure amplitude correctly.

**Figure 12:**
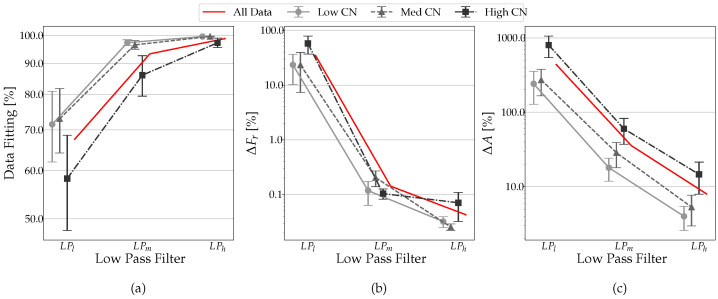
Low-pass trends in the function of the LP filter and CN values: (**a**) data fitting, (**b**) Δ*F*, (**c**) Δ*A*.

We now analyze what influence the condition number CN has on the data fitting and the resonance characteristics measurement combined with the low-pass filter. From the previous plots, we extracted for each column the values of data fitting, Δ*F*, and Δ*A*. We calculated the average value and the error as the CN varies. Analyzing the influence of CN on the data fitting obtained at various LP filter levels, regardless of the various noise levels, we see that values close to 1 of the condition number return higher data fitting values with smaller errors. In Figure 12a, conclusive plots are shown with the trend of the mean value and the errors of obtained data fitting are shown when varying the different values of LP and CN. The average value results are plotted in dashed graphs in terms of CN and MN. It can be seen that an unappropriated condition number worsens the fit of the data by approximately 20% in the case of the low pass filter not being set (Figure 12). Similar results are also found in terms of Δ*F* and Δ*A*, where values of CN close to 1 give better results both in terms of the mean value and error. In Figure 12b,c, conclusive plots are shown with the trend of the mean value and the error of measured frequency and amplitude deviation from the reference when varying the different values of LP and CN.

### 3.2. Zeroing

**Figure 13:**
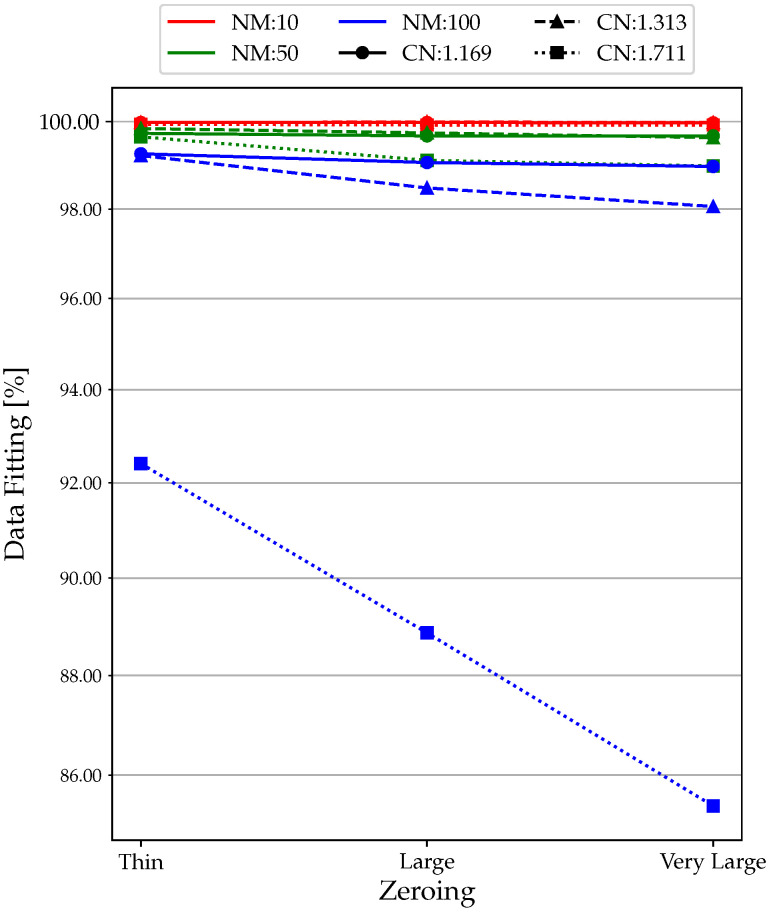
Zeroing analysis: data fitting results.

The effect of zeroing in the measurement of frequency and amplitude of resonance is evaluated by maintaining (in all configurations) the filter LP${}_{h}$, so as to have the best possible input data. From all acquisitions (Figure 13), it is evident that as the area increases, the fitting value decreases. This is due to the fact that as we widen the zeroing zone, it is zeroing out by encompassing non-zero deflection values as we approach the resonance zone.

**Figure 14:**
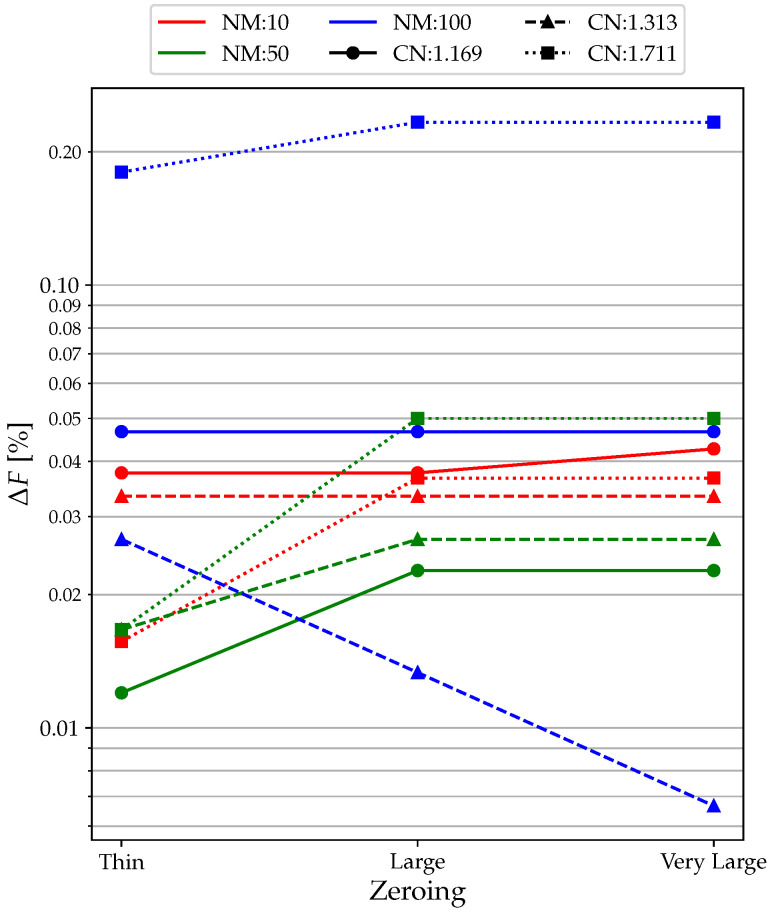
Zeroing analysis:Δ*F* results.

Analyzing the Δ*F* produced by various widths of the zeroing area (Figure 14), it can be seen that as the area increases, the error in the measurement of the resonant frequency increases. This is related to the fact that if the data fitting is lower, the signal reconstruction is worse, and this affects both amplitude and frequency.

Regarding the lower curves, the Δ*F* varies between 0.020 and 0.060%, which is an acceptable range, considering that the resonance frequency is about 30,000 Hz. As for the higher curve, although the operating conditions are worst, there is still an error on the frequency of 0.2% that we can consider acceptable.

In terms of amplitude, by directly analyzing the case and zooming in on the lower areas of the plot, we can see how the influence of the zeroing area is definitely greater. In fact, we go from an error below 2% to about 10%. So it can be argued that, in terms of amplitude variation, it is by no means a parameter to be overlooked.

**Figure 15:**
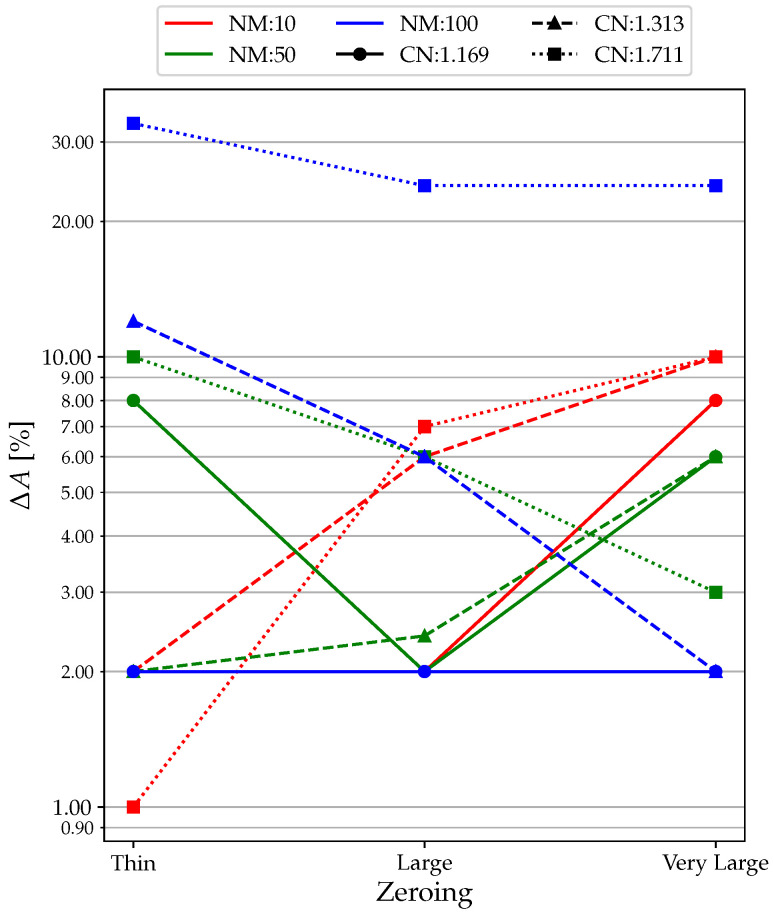
Zeroing Analysis: Δ*A* results.

Analyzing the plots in Figure 15, a trend is observed in which the course of two groups of curves is discordant. In fact, for high NM combined with inappropriate CN, the curves decrease, thus improving Δ*A* with wide zeroing. This phenomenon is still under investigation and will be discussed in future work.

**Figure 16:**
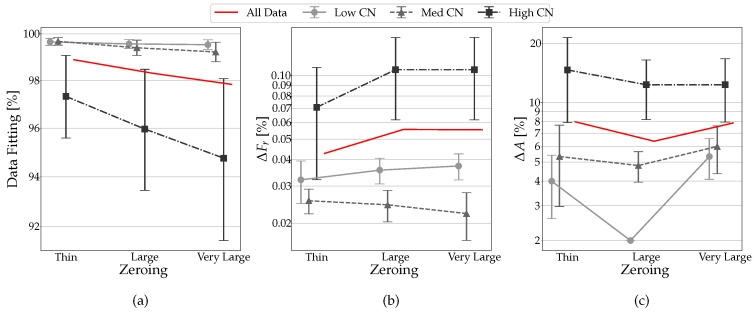
Zeroing trends in functions of zeroing areas and CN values: (**a**) data fitting, (**b**) Δ*F*, (**c**) Δ*A*.

The condition number close to 1 provides a more efficient response: higher zeroing produces a negative difference of approximately 5% in terms of data fitting (Figure 16). In terms of Δ*F*, the increase is contained to about 0.1%, while in terms of amplitude, it goes from 1.8% with CN = 1.169 to 14% with CN = 1.711 and a standard deviation of approximately 18%.

### 3.3. Correlation between Parameters

Regarding the relationship between data fitting and errors in calculating the frequency (Δ*F*) and amplitude (Δ*A*), for the sake of clearness, the data are labeled as follows:

- Color: Blue: NM = 10; Green: NM = 50; Red: NM = 100
- Marker Shape: Circular: CN = Low; Square: CN = Med; Triangular: CN = High
- Marker Filling: Empty: LP${}_{l}$; Pattern: LP${}_{m}$; Full: LP${}_{h}$

**Figure 17:**
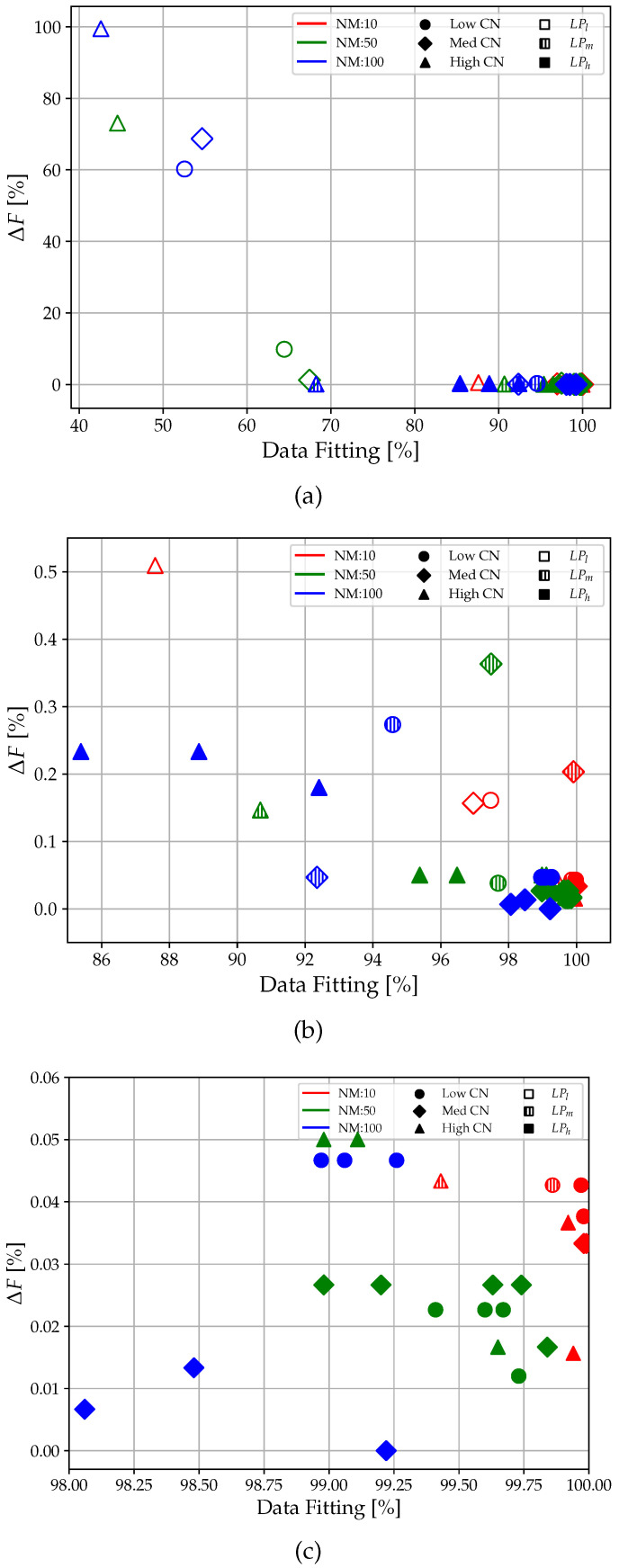
Correlation between the data fitting and the ΔF scatter: the data are related to NM, CN, and LP. Different zoom levels of data fitting are proposed in order to highlight different details, i.e., (**a**) 40–100%, (**b**) 85–100%, (**c**) 98–100%.

**Figure 18:**
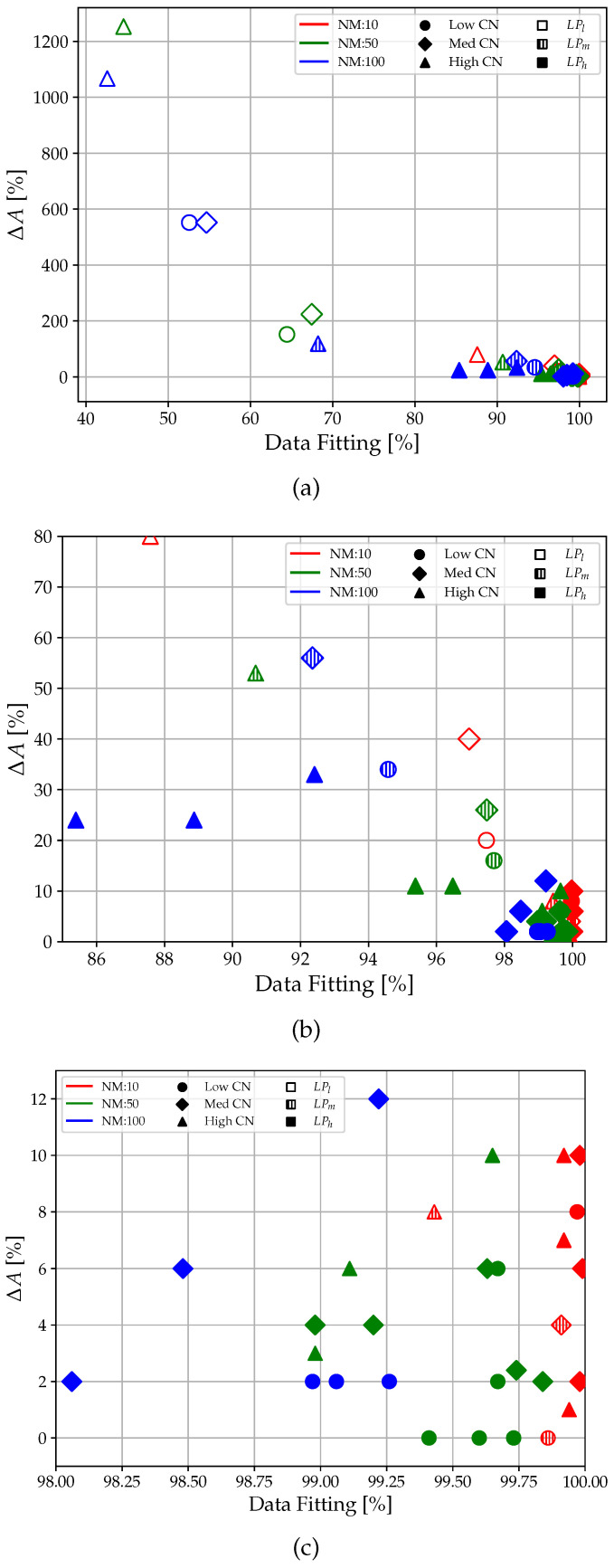
Correlation between the data fitting and the ΔA scatter: the data are related to NM, CN, and LP. Different zoom levels of data fitting are proposed in order to highlight different details: (**a**) 40–100%, (**b**) 85–100%, (**c**) 98–100%.

Figure 17 and Figure 18 present the results at different zoom levels, to make resulting patterns more clear to the reader.

**Figure 19:**
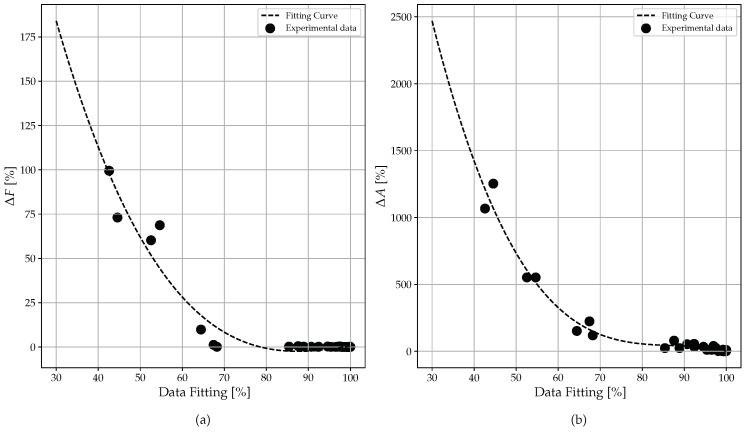
Trends of data fitting against Δ*F* (**a**) and data fitting against ΔA (**b**).

The cases with worse data fits are those with filter LP${}_{l}$, presenting a higher Δ*F* and Δ*A*. As the filtering parameters increase, MN drops, CN tends to one, and the error tends to 0 for data fitting that tends to 100%. In principle, however, as expected, considering all data acquired with no differentiation, data fitting vs. Δ*F* and data fitting vs. Δ*A* (Figure 19) follows a semi-descending parabola trend. This indicates that, as the data fitting increases, the error in the calculation of the frequency (Δ*F*) and the error in the calculation of the amplitude (Δ*A*) decrease.

As a consequence of these results, it is necessary to define a minimum of 95% of data fitting that guarantees good reliability of the results obtained in terms of frequency and amplitude. However, there are very limited cases in which—although the data fitting is high and all of the data control parameters say that we have correctly reconstructed the resonance—we could have some errors in the measurements, even to a low extent. Since this behavior is limited to a small number of cases, it can still be considered acceptable.

## 4. Discussion

Among all of the parameters analyzed, the *low pass filter* (locked to the speed of rotation) significantly impacts the result. The low-pass filter, on average, improves the data fitting from 25% to 30%, decreasing the uncertainty to values close to 0%. The condition number has a significant effect on data fitting as well. Good data fitting indicates that the signal was reconstructed correctly. If the data fitting is high, even if the signal is noisy, acceptable results are still obtained. With the same filtering, there is improvement ranging from 5% to 15%. In terms of frequency, not filtering the data can lead to errors of up to 35%. On the other hand, using a low pass filter almost completely eliminates the uncertainty in the frequency measurement; in terms of the condition number, a high CN can be managed by good filtering, canceling the gap with CN close to 1. This behavior can be explained by considering that the CN is inversely proportional to the noise robustness of the interpolation algorithm, so CN has a stronger effect when noise is present in the acquired signal. In the simulated data, the noise is almost completely filtered, and then the results are improved. In real cases, the noise cannot be removed completely, so it is important to have a good condition number to obtain reliable data. Regarding amplitude, we have the same trend as before for frequency. However, here, the performance gap between filtering with LP${}_{m}$ and LP${}_{h}$ is accentuated. It can be seen that filtering with LP${}_{m}$ has an average uncertainty of 40%, bringing it to values close to 5% with LP${}_{h}$. In terms of amplitude, the condition number has a relevant weight.

With regard to *zeroing* analysis, narrow zeroing is expected to be more efficient than wide zeroing. In fact, a narrow zeroing, in terms of amplitude, keeps the data fitting close to 100%. On the contrary, by widening the zeroing zone, the data fitting descends to values close to 95%. It is fundamental how the CN influences the data fit. With narrow zeroing, the influence of the condition number is less evident. As the zeroing widens, the difference between the good data fitting and the bad one reaches more than 5%. Therefore, we can say that a high CN makes one much more sensitive to the region used for zeroing. We analyze the effect of zeroing in on the results in terms of frequency. The effect is visible between narrow and wide zeroing. Between wide and very wide zeroing, you see less of a difference and the difference tends to flatten out. On average, narrow zeroing lowers uncertainty values by one-third (from 0.06% to 0.04%). However, considering the very low uncertainty values, we can say that the effect of zeroing is almost negligible. The difference between a good and a bad data fit is very clear, almost tripling the uncertainty values under the same conditions In terms of amplitude, we see the same trend as previously seen for frequency. The difference is in the fact that the uncertainty values have constant values between 6% and 8% and, therefore, are not negligible. The data fit that brings the error to values close to 2% versus those of 15% for a bad data fit becomes essential.

Finally, the cross-influence between data fitting, Δ*F*, and Δ*A*, was evaluated. Synthetically, as the data fitting increases, the error decreases, in terms of amplitude and frequency, with quadratic law, as expected.

## 5. Conclusions

In this study, a thorough characterization of blade tip-timing data processing software was performed. For this purpose, a novel methodology for generating synthetic arrival time signals was developed using a mathematical model based on a state-space approach. Synthetic signal arrays were used as the controlled input to characterize the response of generic BTT software under different operating conditions. The sensitivity analysis was carried out under conditions of a single vibration mode and synchronous vibrations. As the state-space approach utilized for controlling the system dynamics proved to be effective, the generated signals were validated through comparison with reference signals. This approach is considered versatile as it can be easily adapted to simulations of any type of vibration. For example, multimodal vibrations or any type of acceleration ramp can be implemented by appropriately modifying the state-space matrices. Future developments will be carried out to implement multiple vibration modes and asynchronous vibration signal generation. 

## Figures and Tables

**Table 1:** A comparison between the imposed and measured resonance frequency ($F_{r}$) and amplitude ($A_{r}$) is shown. The percentage difference between the two is reported ($\Delta F_{r}$ and $\Delta A_{r}$). The resonance amplitude is kept constant at 0.05 mm.

$F_{r}$ [Hz] Imposed	$A_{r}$ [mm] Imposed	$F_{r}$[Hz] Measured	$\Delta F_{r}$ [%] Measured	$A_{r}$ [mm] Measured	$\Delta A_{r}$ [%] Measured
1500	0.05	1500.3	0.02	0.05	0
7500	0.05	7501.5	0.02	0.05	0
15,000	0.05	14,998.4	0.010667	0.05	0
22,500	0.05	22,501.8	0.008	0.051	2
30,000	0.05	29,992	0.026667	0.052	4

**Table 2:** A comparison between the imposed and measured resonance frequency ($F_{r}$) and amplitude ($A_{r}$) is shown. The percentage difference between the two is reported ($\Delta F_{r}$ and $\Delta A_{r}$). The resonance frequency is kept constant at 10,000 Hz.

$F_{r}$ [Hz] Imposed	$A_{r}$ [mm] Imposed	$F_{r}$ [Hz] Measured	$\Delta F_{r}$ [%] Measured	$A_{r}$ [mm] Measured	$\Delta A_{r}$ [%] Measured
10,000	0.001	10,025.4	0.254	0.001	0
10,000	0.005	9999.3	0.007	0.005	0
10,000	0.01	9994.6	0.054	0.01	0
10,000	0.05	9994.6	0.054	0.05	0
10,000	0.1	9994.6	0.054	0.1	0
10,000	0.5	9994.6	0.054	0.501	0.6

**Table 3:** Probe location set used for the test. Each set was calculated for per-rev = 200 and different condition numbers (CN).

CN	Probes Location [°]							
	#1	#2	#3	#4	#5	#6	#7	#8
1	256.19	228.68	227.45	182.09	176.77	138.30	136.89	125.29
1.169	197.97	99.92	91.73	329.36	305.97	286.58	282.24	238.7
1.313	358.77	288.82	286.34	212.70	201.43	86.68	25.49	20.18
1.711	138.19	95.05	86.92	35.92	21.37	14.83	353.41	272.80
